# An Innovative Bulgur Production Method Using Pullulanase Enzyme and Autoclaving–Cooling Cycles to Produce Bulgur with Low Glycemic Index

**DOI:** 10.3390/foods14223972

**Published:** 2025-11-19

**Authors:** Muge Ozbek, Turgay Sanal, Kubra Ozkan, Osman Sagdic, Samuela Palombieri, Francesco Sestili, Hamit Koksel

**Affiliations:** 1 Department of Nutrition and Dietetics, Health Sciences Faculty, Istinye University, Istanbul 34010, Türkiye; dytmugeozbek@gmail.com (M.O.); kubraozkan1907@gmail.com (K.O.); 2 Department of Quality and Technology, Field Crops Central Research Institute, Ankara 06170, Türkiye; turgay.sanal@tarimorman.gov.tr; 3 Department of Food Engineering, Faculty of Chemical and Metallurgical Engineering, Davutpasa Campus, Yildiz Technical University, Istanbul 34349, Türkiye; osagdic@yildiz.edu.tr; 4 Department of Agriculture and Forest Sciences (DAFNE), University of Tuscia, 01100 Viterbo, Italy; palombieri@unitus.it

**Keywords:** bulgur, type3 RS, enzyme modification, gelatinization, retrogradation

## Abstract

Bulgurs with increased resistant starch (RS) contents were produced from high-amylose wheat and Svevo wheat samples by pullulanase treatment and autoclaving–cooling cycles. Cooking properties, color, texture, phenolic content, antioxidant capacity, in vitro glycemic index, and RS contents of bulgurs were examined. RS contents of bulgurs obtained by enzyme application and autoclaving–cooling cycles were 3-folds higher than those of the control bulgurs and reached a level of 9.47%. The GI value of the bulgur produced from high-amylose wheat by pullulanase treatment and autoclaving–cooling cycles was quite low (52.11), and hence it can be classified as a low-GI food. Pullulanase application and autoclaving–cooling cycles had significant effects on textural properties of bulgurs. Phenolic content and antioxidant capacity values were found to be the highest in bulgurs obtained by enzyme application and autoclaving–cooling cycles. These results can be used to improve the health benefits of bulgur by increasing its RS content and decreasing its GI value.

## 1. Introduction

Bulgur is one of the earliest “processed foods”, dating back to prehistoric times, and is usually made from durum wheat [1, 2]. Due to its quick cooking time, relatively lower cost, extended shelf life, and high nutritional value, bulgur is extensively consumed in Türkiye and other Middle Eastern countries [2, 3, 4]. It has been referred to by numerous names throughout history, including cereal, burghul, and burgul [4, 5].

Bulgur is produced from whole kernels through a series of steps: Cleaning, washing, boiling the grains in water, drying them to a safe moisture level for storage, tempering, lightly debranning, cracking, classifying according to particle size, and occasionally polishing [1]. In particular, cooking and drying are two crucial stages in the bulgur production process, as they substantially impact the final product’s quality. Although bulgur is traditionally sun-dried after cooking, hot air drying is widely employed in contemporary bulgur factories [6].

Since bulgur production and consumption are rising in both developed and developing countries, technological advancements in the bulgur production process are crucial for improving its quality [7]. Bulgur has an elevated nutritional value because it is processed less than other cereal products, and the bran layers are usually not removed extensively during processing, resulting in a nourishing product. Bulgur contains higher levels of calcium, iron, and vitamins B1 and B3 compared to other wheat products like pasta and bread [5]. Due to its high fiber content and low glycemic index, bulgur has the potential to lower the risk of various non-communicable diseases [8].

Resistant starch (RS) is defined as the fraction of starch that resists digestion in the small intestine of healthy humans but can be fermented in the large bowel [9]. Because RS cannot be hydrolyzed by enzymes in the small intestine, when it reaches the large intestine, it serves as a fermentation substrate for gut microflora, promoting the growth of beneficial bacteria [10]. When starch is cooked and subsequently cooled, retrogradation takes place, forming RS.

RS is divided into the following 5 groups, according to their chemical and physical properties: RS1, RS2, RS3, RS4, and RS5 [10]. RS1 is resistant to digestion due to its intact structure, and is found in partially ground and unground cereals, while RS2 is granular RS found in raw potatoes and green bananas (ungelatinized). RS3 is retrograded starch that is formed when foods containing starch are cooked and cooled. RS4 is a chemically modified type of resistant starch, while RS5 consists of amylose–lipid complexes [10]. Among these, RS3 is particularly interesting due to its heat stability. Research has demonstrated that RS3 enhances glucose tolerance while lowering blood lipids and plasma cholesterol. Additionally, resistant starch has been observed to improve the intestinal absorption of certain minerals in both humans and rats [9, 11]. Therefore, extensive research has been conducted to obtain high yields of RS3 from various cereal sources. The level of RS3 formation is closely associated with the amylose content of starch, as higher amylose levels correspond to slower digestion rates [11]. Numerous factors—such as amylose content and chain length, autoclaving temperature, and the storage duration and temperature of starch gels—can influence the recrystallization process and, consequently, the formation of RS3 [9]. To generate RS3, starch must first be gelatinized to disrupt its granular structure, followed by retrogradation to allow the starch molecules to recrystallize.

The aim of this study was to enhance the formation of type 3 enzyme-resistant starch in bulgur through debranching and repeated autoclaving–cooling cycles, thereby reducing its glycemic index (GI). The effects of this novel bulgur-processing technique on the cooking, functional, and nutritional properties of bulgur were also examined. Unlike previous research, in which bulgur was not produced using repeated autoclaving–cooling cycles following pullulanase treatment to cleave α-1,6 linkages in starch structure, the present study introduces an innovative and distinctive method for bulgur production.

## 2. Materials and Methods

### 2.1. Materials

Two durum wheat genotypes were used in the study: the Italian cultivar Svevo (SW) and a high-amylose line derived from Svevo (HaW). All reagents, solvents, and Pullulanse enzyme used in this study were obtained from Sigma-Aldrich (St. Louis, MO, USA). Resistant starch and a glucose assay kit were purchased from Megazyme International (Wicklow, Ireland).

### 2.2. Methods

#### 2.2.1. Characterization of Svevo and High-Amylose Wheat Samples

Hectoliter weight was determined with an Ohaus Hectoliter Tester (Chicago, IL, USA), in accordance with Vasiljevic and Banasik [12]. Thousand kernel weight was determined using an electronic kernel counter (Numigral II, Tripette & Renaud, Asnières sur Seine, France), in accordance with the ISO Method (No: 520:2010) [13], using a 20 g clean sample, and the results are given in grams of dry matter. Grain hardness (HI) of the samples was determined with SKCS (Single Kernel Characterization System), performed in accordance with AACC Method No. 55-31.01 (AACCI 2010) [14]. The moisture content of whole meal samples was determined according to AACC International Method No. 44-15.02 (AACCI, 2010) [14]. The protein content of durum wheat and bulgur samples was determined as 5.7 ×N using a LECO FP828 combustion nitrogen analyzer (LECO Instruments Corp., St Joseph, MI, USA) calibrated with EDTA following AACCI Method 46-30.01 (AACCI, 2010) and reported on a dry basis [13]. The ash contents of the samples were determined according to AACCI Standard Method No. 08-01.01 (AACCI, 2010) [14]. Sodium dodecyl sulfate (SDS) sedimentation values (mL) were determined using AACCI Method 56-70 (AACCI, 2010) [14], reported on a 14% moisture basis. The color values of bulgur samples were measured with a colorimeter (Konica Minolta CR-400, Tokyo, Japan) using the L*a*b* color space (CIELAB space). The amount of enzyme–resistant starch content in bulgur samples was determined according to AACCI 32-40.01 (AACCI, 2010) Methods using a glucose assay kit (Megazyme Int., Wicklow, Ireland) [14]. All the analyses were performed at least in triplicate.

#### 2.2.2. Bulgur Production and Resistant Starch Formation

High-amylose wheat sample (HaW) and Svevo wheat (SW) were cleaned using grain cleaning equipment (Quator, Tripette & Renaud, France), and 400 g grain samples were mixed with boiling water at a ratio of 1:2.25 and cooked after the gelatinization of starch was completed (around 55–60 min). The samples were stirred periodically (30 s stirring every 5 min) during cooking, and cooking was terminated when the gelatinization of starch was completed. The cooking time was determined as the time required for the opaque part in the middle of the kernels to disappear when gently squeezed between two glass plates.

Starch modification of cooked durum wheat samples were performed using the method described by Ozturk et al. [9]. For starch modification and other analyses, cooked samples (HaW and SW) were divided into two parts. The cooked wheat samples were autoclaved at 121 °C for 30 min. For the resistant starch modification, 4 mL of pullulanase enzyme (Promozyme 400 PUN/mL, 220 U/kg wheat) was added and incubated at 60 °C for 66 h. After the incubation, the samples were kept in cold storage (4 °C) for 48 h and then autoclaved at 121 °C for 30 min. The cold storage–autoclaving cycle was repeated two more times. Then the samples were dried at 35 °C for 66 h in a drying cabinet (BINDER, Model D-115, Tuttlingen, Germany).

**Figure 1:**
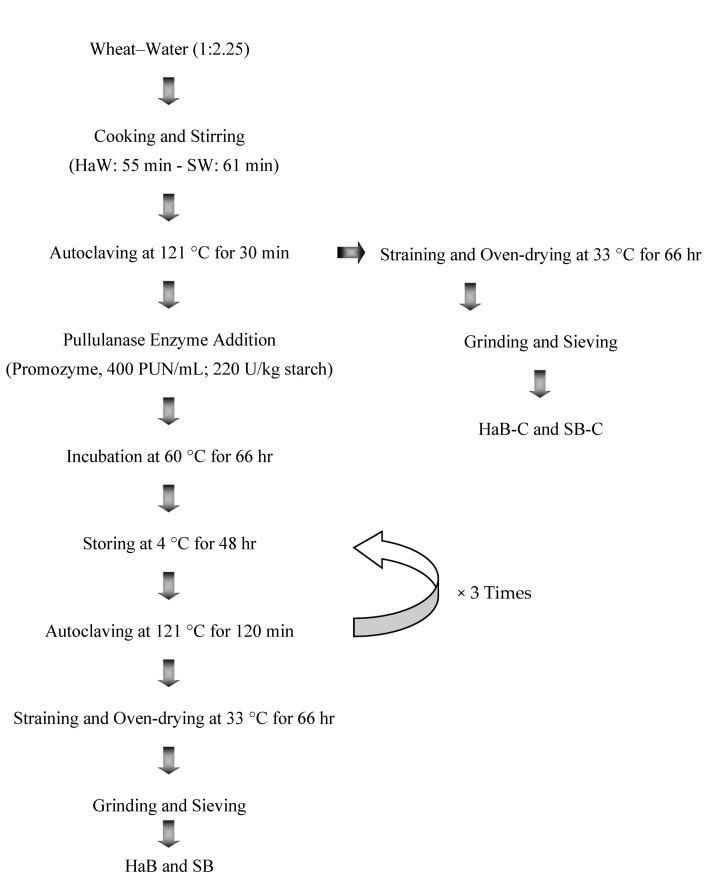
Flow chart of resistant starch production. Haw: high-amylose wheat; SW: svevo wheat; HaB: high-amylose bulgur; SB: Svevo bulgur; HaB-C: high-amylose bulgur-control; and SB-C: Svevo bulgur-control.

The RS3 formation process is shown in Figure 1. A laboratory scale bulgur-grinding machine (Duru Değirmen Makine, Karaman, Türkiye) was used to crush the cooked and modified samples which were then sieved using a 0.5 mm sieve. The product remaining on the sieve was designated as bulgur. Control bulgur samples were produced from Svevo and high-amylose wheat samples without enzyme treatment and autoclaving–cold storage cycles. For the period of this study, the following abbreviations were used for the bulgur samples: Svevo bulgur-control (SB-C), Svevo bulgur (SB), high-amylose bulgur-control (HaB-C), and high-amylose bulgur (HaB).

#### 2.2.3. Determination of the Cooking Properties of Bulgur Samples

The ascertained cooking characteristics of the bulgur samples included the following: weight increase, volume increase, and cooking loss during cooking. The weight and volume increase in the bulgur samples during cooking were determined according to Tekin-Cakmak et al. [1]. To determine the weight gain value, 10 g of bulgur was cooked in 100 mL of boiling water. Upon completion of cooking, excess water from the bulgur samples was removed using filter paper for approximately 5 min, and the samples were weighed [1]. The percentage difference between the pre- and post-cooking bulgur weights was divided by the pre-cooking bulgur weight to determine water absorption.

To determine the volume increase value, a 10 g sample of bulgur was placed in a 250 mL measuring cylinder containing 100 mL of distilled water, and the volume increase was recorded as the dry volume. The same procedures were performed for cooked and drained bulgur, and the volume increase was recorded as the wet volume. The volume increase (%) was calculated by subtracting the dry volume from the wet volume, dividing it by the dry volume, and multiplying it by 100.

A 10 g bulgur sample was cooked in 100 mL of boiling water to determine the cooking loss. After collecting the cooking water in a tarred beaker, it was dried at 98 °C in an air oven. The residue was weighed to calculate the percentage of solid material lost during cooking [15].

#### 2.2.4. Determination of Phenolic Content and Antioxidant Capacity of Bulgur Samples

The extraction of free phenolic compounds (FPCs) and bound phenolic compounds (BPCs) of the samples were performed according to the method earlier described by Shamanin et al. [16]. The Folin–Ciocalteu method modified by Tekin-Cakmak et al. [1] was used to determine concentrations of free and bound phenolics (FPCs and BPCs). 500 μL of Folin–Ciocalteu reagent (2N), 1.5 mL of Na_2_CO_3_ solution (200 g/L), and 7.9 mL of distilled water were combined with 100 μL of methanol extract and left to stand in the dark for 120 min. The absorbance values of the samples were measured at a wavelength of 760 nm using a Shimadzu 150 UV-1800 spectrophotometer (Kyoto, Japan). The results were stated as mg gallic acid equivalent (GAE)/100 g dry weight (dw). The total phenolic content was calculated based on the sum of free and bound phenolic compounds.

DPPH, ABTS, and FRAP tests were used to determine the antioxidant capacity of the samples. The DPPH radical scavenging activity method was used to determine the antioxidant capacity, as described by Singh et al. [17]. Volumes of 0.1 mL of the extracts were mixed with 4.9 mL of a DPPH solution (0.1 mM in methanol). The mixture was incubated at room temperature for 20 min in the dark. After incubation, the samples were measured at a wavelength of 517 nm using a Shimadzu 150 UV-1800 spectrophotometer (Kyoto, Japan).

The ABTS radical cation removal capacity of the samples was determined according to the method described by Re et al. [18], with some modifications. 0.1 mL of the extract was mixed with 2 mL of the diluted ABTS solution. The absorbance values of the samples were measured at 734 nm using a spectrophotometer.

To determine antioxidant capacity, the FRAP method described by Benzie and Strain [19] was used with some modifications. One hundred microliters of the extract was mixed with 900 µL of water and 2 mL of the FRAP reagent and incubated at room temperature for 30 min in the dark. The absorbance values of the samples were measured at 593 nm using a spectrophotometer. The results of all tests performed to determine antioxidant capacity (DPPH, ABTS, FRAP assays) are reported in mg Trolox equivalent (TE)/100 g dw.

#### 2.2.5. Texture Profile Analysis of Bulgur Samples

Texture profile analysis (TPA) of cooked bulgurs were performed using a Texture Analyzer (TA.XT2 Plus, Stable Micro System Ltd., Surrey, UK) according to Tekin-Cakmak et al. [1]. Before analysis, 10 g of the weighed bulgur samples were cooked with 150 mL of boiled water. The samples were placed in a metal ring with a diameter of 80 mm and a height of 50 mm, located in the center of the equipment’s test platform to ensure they had a standard shape; a two-cycle compression process was applied to the sample. Speeds were set at 1 mm/s for the pre- and post-test stages. In each cycle, the samples were compressed up to 4 mm using a P/36 flat cylindrical probe. The triggering force between compressions was set to 5 g, and the time between compressions was set to 5 s.

#### 2.2.6. In Vitro Glycemic Index Value of Bulgur Samples

In vitro starch hydrolysis index (HIn) and glycemic index (GI) values of cooked bulgur samples were determined using a glucose assay kit (Megazyme Int., Wicklow, Ireland) following the protocols established by Goñi et al. [20]. 100 mg of the cooked bulgur samples was placed in 50 mL centrifuge tubes containing ten glass beads (5 mm diameter). Two milliliters of 0.05 M HCl containing pepsin (5 mg/mL; Sigma, P7000) were added, and the tubes were incubated in a shaking water bath at 37 °C for 30 min to simulate gastric digestion. Subsequently, 4 mL of sodium acetate buffer (0.5 M, pH 5.2), 1 mL of an enzyme solution containing pancreatin (0.104 g; Sigma, P7545), and amyloglucosidase (14.45 U; 3300 U/mL; Megazyme, Ireland) were added to each tube. The mixtures were then incubated horizontally in a shaking water bath at 37 °C to simulate intestinal digestion. Aliquots (100 μL) were collected at 0 and 90 min of incubation and mixed immediately with 1 mL of absolute ethanol to inactivate the enzymes. The samples were centrifuged at 800× g for 10 min, and the glucose concentration in the supernatant was determined using the glucose oxidase–peroxidase (GOPOD) reagent (Megazyme, Ireland). Absorbance was measured at 510 nm using a spectrophotometer.

#### 2.2.7. Statistical Analysis

All experiments were conducted in triplicate, and the findings were presented as the mean ± standard deviation. Statistical analysis was conducted using SPSS Statistics Software (IBM version 22, Armonk, NY, USA). A one-way ANOVA followed by Tukey’s post hoc test was applied to evaluate the differences among the bulgur samples (*p* < 0.05). The differences between physical and chemical analysis of SW and HaW samples were evaluated by a *T*-test (*p* < 0.05).

## 3. Results and Discussion

### 3.1. Physical and Chemical Analysis of Wheat Samples

Physical and chemical analysis results of SW and HaW samples are given in Table 1. HaW had a significantly lower hectoliter weight (73.07 kg/hl) than SW (81.00 kg/hl) (*p* < 0.05). Similarly, the thousand kernel weight of HaW (31.30 g) was significantly lower than that of SW (41.55 g) (*p* < 0.05). Previous studies have reported the hectoliter weight of Svevo durum wheat as 79.90 kg/hl [21]. The thousand kernel weight of Svevo durum wheat has been found to be 38.12 g [21], which is consistent with the results of the present study.

Grain hardness is a key physical property in determining the suitability of wheat for different end-use products and plays a crucial role in processing conditions, such as tempering and milling. The hardness index of SW (88.0%) was found to be significantly (*p* < 0.05) higher than that of HaW (71.05%). Canay-Unsal et al. [21] reported a hardness value of 86.16% for Svevo grain. Hence, the hardness values of Svevo and high-amylose wheat samples in the current study are close to the ones found in the literature. The grain diameter of HaW (2.95 mm) was significantly smaller than that of SW (3.09 mm) (*p* < 0.05). SDS sedimentation and yellowness values (b*) did not differ significantly between SW and HaW.

The protein content of SW was significantly (*p* < 0.05) lower than that of HaW. Protein content is an important parameter in the evaluation of wheat quality and is influenced by both genotype and environmental conditions. A previous study has reported protein content in SW as 14.85% [21], which is slightly higher than the values observed in this study.

SDS sedimentation is an important parameter used in the evaluation of protein quantity and quality and, like protein content, it is affected by genotype and environmental factors [22]. The SDS sedimentation values for SW and HaW were found to be 29 mL and 34 mL, respectively, indicating a difference in gluten quality between the two wheat types. However, both samples showed satisfactory gluten quality. Typically, higher SDS sedimentation values are associated with stronger gluten networks and better protein quality, which are desirable traits for wheat-based products like pasta and bread [23]. The higher value in HaW compared to SW can be attributed to its higher protein content, as reported in Table 1 [24]. Canay-Unsal et al. [21] reported an SDS sedimentation value of 16 mL for their Svevo wheat sample. The higher SDS sedimentation values observed in the present study compared to those of Svevo and other durum wheat genotypes in the literature may be attributed to differences in growing season and location [25]. Svevo durum wheat variety with high yellow pigmentation is an important prevailing cultivar in Southeast Anatolia and is used in bulgur production [26]. The yellowness values (b*) were found to be 26.17 in SW and 26.82 in HaW, both of which are relatively higher compared to the values reported by Savas and Basman [15].

### 3.2. Color Properties of Bulgur Samples

The final color of bulgur is significantly affected by the color of the raw material and the processes (e.g., cooking, drying, sorting, and grinding) used in bulgur production [27]. Consumers generally prefer bulgur with a bright yellow hue [6].

**Figure 2:**
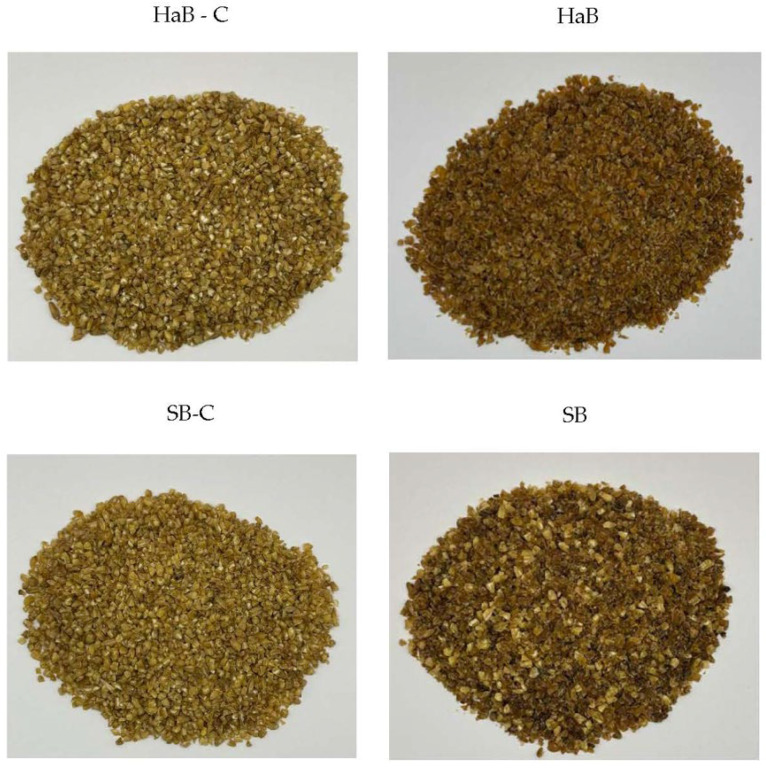
Photographs of the bulgur samples. HaB-C: high-amylose bulgur-control; HaB: high-amylose bulgur; SB-C: Svevo bulgur-control; SB: Svevo bulgur.

In this study, treatment with pullulanase enzyme followed by autoclaving–cooling cycles decreased the L* and b* values, but increased the a* value significantly in both samples compared to their respective controls (*p* < 0.05) (Table 2). Instead, the genotype difference did not affect the color of both the control bulgurs, except for the b* parameter, which is significantly lower in HaB compared to SB. Similar results were reported by Yilmaz and Koca [27], who observed that the combination of autoclave cooking and microwave drying decreased L* and b* values while increasing a* values in durum bulgur, negatively impacting its overall color characteristics. It was stated that the application of heat treatment at high temperatures during bulgur production results in negative effects, such as darkening of the color in bulgur. This is mainly caused by the Maillard reaction, which occurs between reducing sugars and amino groups of proteins/amino acids in bulgur upon heat treatment [28]. Additionally, Dueck et al. [3] noted that processing conditions can significantly affect the brightness of bulgur. Previous studies have shown that the autoclave cooking method caused color darkening in bulgur samples compared to traditional and microwave cooking methods [6, 27]. In addition to cooking methods, drying temperature plays a crucial role in determining bulgur quality. Exposure to high temperatures during drying can cause dulling and darkening of the bulgur color [26]. The photographs of the bulgur samples are given in Figure 2.

### 3.3. Cooking Properties of Bulgur Samples

Cooking characteristics of bulgur samples (weight increase, volume increase, and cooking loss) are given in Table 2. Short cooking time and relatively higher water absorption capacity are desired bulgur characteristics by consumers [1, 27]. The weight increase (%) of the bulgur samples varied significantly among the different treatments and genotypes. The SB sample (regular durum wheat) showed the highest weight increase (195.70%) which was significantly higher than the respective control sample (SB-C, 164.25%), indicating that enzyme treatment and autoclaving–cooling cycles increased water absorption capacity (Table 2). This may be due to the structural changes that support higher hydration in starch and protein matrices in regular wheat. On the other hand, the HaB (high-amylose durum wheat) showed the lowest weight increase (158.87%) and even a lower weight increase than the respective control sample (HaB-C, 175.71%). When comparing the control samples, the slightly higher weight increase in HaB-C compared to SB-C suggests that the untreated high-amylose wheat genotype may initially absorb more water. However, after enzymatic treatment and autoclaving–cooling cycles, HaB absorbed significantly less water than SB, reversing this trend. This indicates that the effects of processing vary, depending on the starch composition of the genotype, especially amylose content. Hendek-Ertop [29] stated that the weight increase values of industrial and homemade einkorn bulgur samples were 149% and 151%, respectively, while durum bulgur exhibited a higher weight increase value (180%) compared to einkorn bulgur [29]. The differences might be attributed to variations in grain properties and kernel size among wheat samples.

The volume increase values of SB-C, SB, HaB-C and HaB samples were 238.10%, 268.80%, 206.25%, and 227.68%, respectively. In both genotypes, pullulanase treatment followed by autoclaving–cooling cycles led to an increase in volume, with SB-C and HaB-C showing similar values, as well as SB and HaB samples. Yilmaz and Koca [27] found the volume increase value of the autoclaved and hot air-dried durum bulgur samples was 200.00%, while einkorn bulgur produced under the same conditions had a lower increase (141.67%). It was reported that durum bulgur required a longer cooking time than einkorn bulgur, allowing it to absorb more water and expand more significantly. A lower volume increase indicates reduced water absorption, resulting in a firmer texture after cooking. Therefore, it is desirable to have a higher volume increase in bulgur. The results of this study suggest that autoclaving–cooling cycles had a minimal impact on improving volume increase.

Cooking loss values of SB-C, SB, HaB-C, and HaB samples were determined as 6.45%, 18.3%, 6.9%, and 15.75%, respectively. Pullulanase enzyme treatment and autoclaving–cooling cycles significantly increased the cooking loss in both wheat genotypes compared to their controls (*p* < 0.05). Although higher cooking loss values are generally undesirable for bulgur and pasta, bulgur is typically not cooked in excess water and residual water is not removed after cooking, meaning that soluble nutritional constituents are not lost through draining. Hence, higher cooking loss values are unlikely to be a major quality problem for the consumer and will not result in loss of soluble nutritional constituents from the cooking water.

### 3.4. Texture Profiles of Bulgur Samples

Hardness, adhesiveness, springiness, cohesiveness, chewiness and resilience values of the bulgur samples are given in Table 3. Hardness, defined as the force exerted by the sample against the probe during the first compression, is a crucial quality parameter in bulgur, as excessively low or high values are undesirable [27]. Hardness values of SB-C, SB, HaB-C, and HaB samples were determined as 588.22, 447.54, 517.74, and 293.61 g, respectively. The results indicate that both wheat genotype and processing treatments (pullulanase enzyme treatment and autoclaving–cooling cycles) significantly (*p* < 0.05) influenced the hardness of the bulgur samples. Before treatment (control samples), the HaB-C had a lower hardness compared to SB-C, indicating that the high-amylose content resulted in a softer texture. This may be due to differences in starch composition, as high-amylose starch may form a less cohesive structure, leading to reduced hardness. After treatment, the difference between the genotypes became more pronounced, with HaB showing the lowest hardness, which was significantly lower than that of SB. This suggests that the structural changes induced by processing (enzyme treatment and autoclaving–cooling cycles) had a greater softening effect on high-amylose wheat than on Svevo wheat.

Yilmaz and Koca [27] found the hardness values of durum and einkorn bulgur samples obtained by autoclaving and drying in hot air to be 16,802.98 g and 24,612.30 g, respectively. These values are notably higher than the ones in the present study. The reason for the reduced hardness values of the bulgur samples might be due to the repeated autoclaving–cooling cycles. Besides this, the probe (50 mm) used in texture analysis by Yilmaz and Koca [27] had a larger diameter than the one (32 mm) used in the present study.

Bulgur is a coarsely ground product. Its particle size, after boiling, drying, and grinding, is usually >1.5 mm, indicating that anatomic parts of kernels are not destroyed. Hence heat treatment conditions (e.g., boiling, autoclaving) may affect the integrity of the cells in bulgur particle. This might in turm influence the textural properties of cooked bulgur. Batista et al. [30] reported that unprocessed beans had the highest hardness values. However, the hardness of autoclaved beans was approximately reduced in the range of 4–49%, depending on the autoclaving conditions, indicating that the combined effect of pressure and temperature probably increased the cell separation, leading to lower hardness.

Adhesiveness is defined as the negative force required to pull the probe away from the sample. High levels of adhesiveness in products cause sensory and visual problems for consumers [27]. Adhesiveness values of cooked SB-C, SB, HaB-C, and HaB samples were determined as −6.49, −3.22, −6.65, and −3.00 g × s, respectively. Pullulanase enzyme treatment and autoclaving–cooling cycles decreased the adhesiveness significantly in both wheat samples compared to their controls (*p* < 0.05), indicating a notable improvement in this parameter. Adhesiveness in cooked bulgur is caused by the starch chains leaching out from the inner part of the cooked particle. Autoclaving–cooling cycles after debranching tends to decrease adhesiveness, possibly by RS formation via retrogradation of these individual starch chains on the surface of bulgur particles. After repeated autoclaving–cooling cycles, bulgur particles become less sticky and more separated, since the starch chains on their surfaces associate with each other, forming higher levels of RS which does not lead to an adhesive surface. Quite high RS3 levels (>9%) obtained in the present study also support this view. Yilmaz [31] reported an adhesiveness value of −16.80 g × s for durum bulgur processed using autoclave cooking followed by hot air drying. This value is higher than those observed in the present study, likely due to differences in probe diameter and bulgur production methods.

The springiness values are defined as the elasticity of the samples, which are calculated by the ratio of the time (distance) between the second and first compression during the analysis [31]. The springiness values of SB and HaB samples (0.86 and 0.96) were significantly (*p* < 0.05) higher than those of the control samples (0.78 and 0.66). Yilmaz and Koca [27] found that the durum bulgur samples produced by the autoclave cooking and hot air drying method had higher springiness values compared to the ones produced by the microwave cooking and hot air drying method. Autoclaving application used in the present study seems to have an increasing effect on the springiness value, as already observed by Yılmaz and Koca [27].

Cohesiveness is described as the degree to which a substance is compressed between the teeth before breaking. Cohesiveness values of SB and HaB samples (0.66 and 0.59) were significantly (*p* < 0.05) higher than those of the respective control samples (0.45 and 0.46). There were no drastic changes in cohesiveness values with autoclaving and cooling cycles and also with the utilization of high-amylose wheat. The results are in line with the reported cohesiveness values of 0.48 for Kızıltan bulgur by Tekin-Cakmak [1] and 0.56 for durum bulgur by Yilmaz and Koca [27].

Chewiness represents the energy required to chew solid foodstuffs and is obtained by multiplying hardness, cohesiveness, and springiness values. The chewiness values of SB-C, HaB-C, SB, and HaB samples were 265.36, 233.41, 163.02, and 130.33, respectively. It is noteworthy that chewiness values of SB and HaB samples are significantly lower than those of the respective control bulgur samples (*p* < 0.05). The lower chewiness value indicates that the food can be chewed more easily, which is a desirable characteristic for bulgur. As expected, lower hardness values in bulgur samples will have a decreased effect on chewiness [31]. Yilmaz and Koca [27] stated that autoclave cooking and microwave drying methods negatively affected (increased) chewiness in durum bulgur samples compared to other cooking and drying methods. On the other hand, in the present study, autoclaving–cooling cycles improved (decreased) the chewiness values of the bulgur samples.

Resilience is expressed as the ability of a foodstuff to retrieve its original shape after removing a deforming force. The resilience values of SB-C, HaB-C, SB, and HaB samples were 0.25, 0.27, 0.38, and 0.36, respectively. Pullulanase enzyme treatment and autoclaving–cooling cycles significantly increased the resilience value in both bulgur samples compared to the respective control samples (*p* < 0.05).

Autoclaving–cooling cycles applied to obtain bulgur with high RS3 values increased the adhesiveness, springiness, cohesiveness, and resilience values of bulgur samples, while decreasing the chewiness and hardness values. This is thought to be due to the application of more than one autoclaving–cooling cycle as well as pullulanase treatment. The autoclaving–cooling cycles and pullulanase treatment led to major changes in starch molecular structure and recrystallization which are expected to influence the textural properties to a great extent.

### 3.5. Phenolic Contents and Antioxidant Capacity of Bulgur Samples

Phenolic content and antioxidant capacity values of bulgur samples were determined as free and bound forms (Table 4). Pullulanase enzyme treatment and autoclaving–cooling cycles had an increasing effect on free, bound, and total phenolic contents in both samples compared to the respective control (*p* < 0.05). This is probably due to increased extractability of phenolic compounds after autoclaving–cooling cycles. Combined application of enzyme hydrolysis and autoclaving–cooling cycles could also affect extractability of phenolic compounds. The difference in the quantity of phenolic compounds in the bulgur samples used in the study can be attributed to the raw material properties of the samples and the differences in the bulgur production process. The bound phenolic contents of the bulgur samples were found to be higher than the free phenolic contents. These results agree with a previous study by Tekin-Cakmak et al. [1], highlighting that phenolic compounds in wheat grain are mostly in bound form due to covalently binding to cell wall polysaccharides.

DPPH, ABTS, and FRAP tests were used to evaluate the antioxidant properties of the samples (Table 4). Pullulanase enzyme treatment and autoclaving–cooling cycles increased the antioxidant capacity significantly in free and bound forms determined by all tests (ABTS, FRAP, and DPPH) compared to their respective controls (*p* < 0.05). In the present study, the different antioxidant activities of the two bulgur samples produced from the same wheat sample are probably due to the difference in bulgur production processes. The results are in line with the previous studies indicating that thermal treatments, especially autoclaving, had an improving effect on phenolic content and antioxidant activity of wheat and oat bran [32], wheat bran [33], and bulgur [8]. Increased antioxidant activity might be due to increased extractability of antioxidant compounds after autoclaving–cooling cycles were applied to increase RS3 content.

### 3.6. Resistant Starch Contents of Bulgur Samples

Foods that contain resistant starch (RS) have the potential to reduce some non-communicable diseases, including diabetes, obesity, and colon cancer. A correlation between the amount of amylose in the kernel and the content of resistant starch in derived foods has been ascertained [34]. RS contents of bulgur samples varied between 2.35% and 9.47% (Table 5). The RS content of SB and HaB bulgur samples obtained by pullulanase enzyme treatment and autoclaving–cooling cycles were 9.27% and 9.47%, respectively, while the RS contents of the corresponding control bulgur samples were 2.35% and 3.24%, respectively. A total of 15 commercial bulgur samples representing 5 brands from different retailers in Türkiye were found to have total resistant starch contents between 2.1% and 2.8% [35]. These results are in line with the RS contents of control bulgur samples in the present study. Wang et al. [36] stated that if foods are cooked and stored at cool temperatures, their resistant starch levels will be higher compared to foods that have not been treated the same way [36].

The bulgur produced from the high-amylose wheat (HaW) sample through pullulanase treatment followed by autoclaving–cooling cycles had a significantly higher RS content (*p* < 0.05) compared to the bulgur produced from regular wheat (SW) using the same procedure. This is probably due to the differences in their amylose content. The bulgur samples with increased RS contents were subjected to three autoclaving–cooling cycles after the pullulanase enzyme treatment. During the autoclaving–cooling cycles and drying stage, starch molecules undergo degradation, forming tightly packed structures stabilized by hydrogen bonds. This structural rearrangement reduces the accessibility of starch to digestive enzymes [9].

Consistent with these findings, Ashwar et al. [37] reported that rice varieties subjected to two autoclaving–retrogradation cycles exhibited significantly higher RS contents than their native starch counterparts. The autoclaving cooking method increases the resistant starch content in direct proportion to the amylose content [38]. Hence, the innovative treatments used in the present study for bulgur production had a significant increasing effect on resistant starch content. To the best of the authors’ knowledge, this is the first study to implement repeated autoclaving–cooling cycles after pullulanase treatment in bulgur production. This innovative approach resulted in an approximately three-fold increase in RS content in bulgur samples.

### 3.7. Estimated Hydrolysis Index and Glycemic Index Values of Bulgur Samples

The estimated hydrolysis index (HIn) and glycemic index (GI) values of cooked and freeze-dried bulgur samples are shown in Table 5. Hydrolysis index values ranged from 22.58 to 59.91. The bulgur samples (SB and HaB) treated with pullulanase enzyme and subjected to autoclaving–cooling exhibited significantly lower HIn values than their respective controls (*p* < 0.05). Foods are classified based on their GI as low (GI ≤ 55), medium (GI 56–69), and high (GI ≥ 70) [39]. SB and its control bulgur had GI values of 62.30 and 72.60, respectively, while HaB and its control bulgur had GI values of 52.11 and 70.29. The present study successfully achieved the highest RS3 levels in bulgur reported to date, along with one of the lowest glycemic index values in the literature for bulgur.

The GI values of SB and HaB samples treated with pullulanase and autoclaving–cooling cycles were significantly lower than those of their control groups (*p* < 0.05), classifying SB as a medium-GI food and HaB as a low-GI food. This demonstrates the potential to reduce the GI of bulgur through pullulanase treatment and autoclaving–cooling cycles, likely due to increased resistant starch (RS) content. Carbohydrates typically raise blood glucose levels approximately 15–45 min after consumption. However, foods rich in RS pass undigested into the large intestine, leading to a slower and more controlled rise in blood glucose [40]. Autoclaving and pullulanase treatment increased the extractability of free, bound, and total phenolic compounds in SB and HaB bulgurs compared to their respective controls (SB-C and HaB-C). This increase was accompanied by corresponding decreases in their GI. The results indicate that higher level of extractable phenolic compounds might have a reducing effect on starch digestibility and GI values.

The significantly lower GI of HaB (52.11) compared to SB (62.30) may be attributed to its higher amylose content. Foods with high-amylose levels tend to produce more RS upon processing, thereby reducing GI values [36]. Similarly, Zheng et al. [41] found that autoclaved rice varieties exhibited lower Hin and GI values than their non-autoclaved counterparts, aligning with the findings of this study.

Several studies suggest that the GI values of foods decrease with the addition of resistant starch. Ma and Lee [42] reported that muffins supplemented with 30 g of RS lowered postprandial glycemic responses in sedentary individuals with abdominal obesity. Further research is needed to confirm the GI-lowering effects of bulgur produced from high-amylose wheat on human health. There are currently no official guidelines for regulating resistant starch intake, but some studies suggest that consuming 6–12 g of resistant starch per meal may be beneficial for postprandial glucose and insulin levels. Furthermore, estimates of daily RS intake in Europe and Australia range from 3 to 6 g [43].

## 4. Conclusions

Healthy eating habits, typical of a Mediterranean diet, frequently consist of cereal-based meals. The MEDWHEALTH project supported by PRIMA aims to enhance the health benefits of staple foods (flat breads, pasta, and bulgur) and increase their healthfulness.

Although cereals such as bread wheat, barley, corn, and einkorn are used in bulgur production, durum wheat is widely preferred. It is rich in dietary fiber and contains various vitamins and minerals. Bulgur production with enzyme application and autoclaving–cooling cycles increased RS content (9.27%, 9.47%) and decreased HIn content (41.16, 22.58) and GI (62.30, 52.11) values compared to control bulgurs produced using conventional methods. Pullulanase treatment and autoclaving–cooling cycles increased the phenolic content and antioxidant capacity values (evaluated using DPPH, ABTS, and FRAP tests) of the bulgur samples. The pullulanase enzyme treatment and autoclaving–cooling cycles decreased the hardness and chewiness values but increased the springiness, cohesiveness and resilience values compared to the respective bulgur control sample.

It can be concluded that high-amylose durum wheat can be used for the production of bulgur with better nutritional properties, especially higher resistant starch content and lower glycemic index values by pullulanase enzyme treatment and autoclaving–cooling cycles.

## Figures and Tables

**Table 1:** Physical and chemical analysis of wheat samples.

Samples	HW (kg/hL)	TKW (g/db)	HI (%)	Diameter (mm)	Protein (Nx5.7, %)	SDS (mL)	b*
SW	81.00 ± 0.05 ^a^	41.55 ± 0.50 ^a^	88. 00 ± 1.06 ^a^	3.09 ± 0.024 ^a^	12.71 ± 0.08 ^b^	29.0 ± 1.41 ^a^	26.17 ± 0.08 ^a^
HaW	73.07 ± 0.07 ^b^	31.30 ± 0.14 ^b^	71.05 ± 0.57 ^b^	2.95 ± 0.03 ^b^	13.07 ± 0.08 ^a^	34.0 ± 1.41 ^a^	26.82 ± 0.55 ^a^

SW: Svevo wheat; HaW: high-amylose wheat sample; db: dry basis; HW: hectoliter weight; TKW: thousand kernel weight; HI: hardness index; SDS: sodium dodecyl sulfate sedimentation; b*: yellowness value. Values followed by different letters in the same column are significantly different (*p* < 0.05).

**Table 2:** Cooking and color properties of bulgur samples.

Samples	Weight Increase (%)	Volume Increase (%)	Cooking Loss (%)	L*	a*	b*
SB-C	164.25 ± 0.95 ^c^	238.10 ± 4.76 ^ab^	6.45 ± 0.07 ^b^	52.07 ± 0.97 ^a^	4.42 ± 0.20 ^b^	25.46 ± 0.35 ^a^
HaB-C	175.71 ± 2.71 ^b^	206.25 ± 6.25 ^b^	6.90 ± 0.28 ^b^	53.27 ± 0.95 ^a^	4.79 ± 0.38 ^b^	24.64 ± 0.83 ^a^
SB	195.70 ± 1.20 ^a^	268.80 ± 31.3 ^a^	18.30 ± 0.28 ^a^	39.20 ± 0.90 ^b^	6.56 ± 0.30 ^a^	18.65 ± 0.33 ^b^
HaB	158.87 ± 6.73 ^c^	227.68 ± 15.18 ^a b^	15.75 ± 1.77 ^a^	39.41 ± 0.71 ^b^	6.84 ± 0.25 ^a^	15.90 ± 0.73 ^c^

SB-C: Svevo bulgur-control; SB: Svevo bulgur; HaB-C: high-amylose bulgur-control; HaB: high-amylose bulgur. Values followed by different letters in the same column are significantly different (*p* < 0.05).

**Table 3:** Texture profiles of bulgur samples.

Samples	Hardness (g)	Adhesiveness (g × s)	Springiness	Cohesiveness	Chewiness	Resilience
SB-C	588.22 ± 7.40 ^a^	−6.49 ± 0.25 ^b^	0.78 ± 0.01 ^c^	0.45 ± 0.03 ^c^	265.36 ± 4.10 ^a^	0.25 ± 0.04 ^b^
HaB-C	517.74 ± 5.83 ^b^	−6.65 ± 0.36 ^b^	0.66 ± 0.03 ^d^	0.46 ± 0.02 ^c^	233.41 ± 2.43 ^b^	0.27 ± 0.01 ^b^
SB	447.54 ± 5.08 ^c^	−3.22 ± 0.16 ^a^	0.86 ± 0.01 ^b^	0.66 ± 0.01 ^a^	163.02 ± 3.29 ^c^	0.38 ± 0.01 ^a^
HaB	293.61 ± 3.56 ^d^	−3.00 ± 0.10 ^a^	0.96 ± 0.03 ^a^	0.59 ± 0.02 ^b^	130.33 ± 3.07 ^d^	0.36 ± 0.01 ^a^

SB-C: Svevo bulgur-control; SB: Svevo bulgur; HaB-C: high-amylose bulgur-control; HaB: high-amylose bulgur. Values followed by different letters in the same column are significantly different (*p* < 0.05).

**Table 4:** Free, bound phenolic content and antioxidant capacity of bulgur samples.

	**Phenolic Content (mg GAE/100 g db)**		
**Samples**	Free	Bound	Total
SB-C	264.22 ± 1.74 ^d^	285.42 ± 0.81 ^d^	549.64 ± 1.42 ^d^
HaB-C	280.03 ± 0.71 ^c^	302.81 ± 1.27 ^c^	582.85 ± 0.59 ^c^
SB	287.95 ± 1.74 ^b^	325.99 ± 2.03 ^b^	613.94 ± 0.29 ^b^
HaB	295.50 ± 1.44 ^a^	391.38 ± 2.12 ^a^	686.88 ± 3.20 ^a^
	**ABTS (mg TE/100 g db)**		
	Free	Bound	Total
SB-C	117.86 ± 0.46 ^d^	124.55 ± 0.47 ^d^	242.41 ± 0.42 ^d^
HaB-C	122.12 ± 0.48 ^c^	132.96 ± 0.48 ^c^	255.08 ± 0.84 ^c^
SB	125.16 ± 0.47 ^b^	184.50 ± 3.05 ^b^	309.67 ± 2.94 ^b^
HaB	130.74 ± 0.32 ^a^	190.17 ± 0.98 ^a^	320.91 ± 1.29 ^a^
	**DPPH (mg TE/100 g db)**		
	Free	Bound	Total
SB-C	27.19 ± 1.04 ^c^	79.45 ± 1.04 ^d^	106.64 ± 1.04 ^d^
HaB-C	38.87 ± 0.70 ^b^	85.17 ± 0.70 ^c^	124.03 ± 1.22 ^c^
SB	36.47 ± 0.39 ^b^	148.22 ± 0.68 ^b^	184.69 ± 1.04 ^b^
HaB	49.95 ± 1.42 ^a^	152.83 ± 0.71 ^a^	202.78 ± 1.23 ^a^
	**FRAP (mg TE/100 g db)**		
	Free	Bound	Total
SB-C	18.92 ± 0.58 ^d^	53.53 ± 0.45 ^c^	18.92 ± 0.58 ^d^
HaB-C	23.91 ± 0.57 ^c^	55.19 ± 0.81 ^b^	23.91 ± 0.57 ^c^
SB	26.63 ± 0.67 ^b^	69.68 ± 0.33 ^a^	26.63 ± 0.67 ^b^
HaB	28.44 ± 0.68 ^a^	70.19 ± 0.46 ^a^	28.44 ± 0.68 ^a^

SB-C: Svevo bulgur-control; SB: Svevo bulgur; HaB-C: high-amylose bulgur-control; HaB: high-amylose bulgur; ABTS 2,2′-azino-bis (3-ethylbenzothiazoline6- sulphonic acid); DPPH 2,2-diphenyl-1-picrylhydrazyl radical scavenging activity; FRAP Iron (III) Reducing Antioxidant Power. Values followed by different letters in the same column are significantly different (*p* < 0.05).

**Table 5:** Resistant starch content and estimated HIn and GI values of bulgur samples.

Samples	RS (%)	HIn	GI
SB-C	2.35 ± 0.04 ^d^	59.91 ± 0.50 ^a^	72.60 ± 0.28 ^a^
HaB-C	3.24 ± 0.02 ^c^	55.71 ± 0.19 ^b^	70.29 ± 0.10 ^b^
SB	9.27 ± 0.03 ^b^	41.16 ± 0.56 ^c^	62.30 ± 0.31 ^c^
HaB	9.47 ± 0.02 ^a^	22.58 ± 1.13 ^d^	52.11 ± 0.62 ^d^

SB-C: Svevo bulgur-control; SB: Svevo bulgur; HaB-C: high-amylose bulgur-control; HaB: high-amylose bulgur; HIn: hydrolysis index; GI: glycemic index. Values followed by different letters in the same column are significantly different (*p* < 0.05).

## Data Availability

The original contributions presented in this study are included in the article. Further inquiries can be directed to the corresponding authors. Our data will be made available upon request.
